# XRCC3 and RAD51 Expression Are Associated with Clinical Factors in Breast Cancer

**DOI:** 10.1371/journal.pone.0072104

**Published:** 2013-08-20

**Authors:** Jia Hu, Ning Wang, Ya-Jie Wang

**Affiliations:** 1 The Second Department of Oncology, The First Affiliated Hospital of Chinese PLA General Hospital, Beijing, P.R. China; 2 Department of Oncology, Changhai Hospital, Second Military Medical University, Shanghai, P.R. China; Health Canada and University of Ottawa, Canada

## Abstract

**Aims:**

XRCC3 and RAD51 are two important members in homologous recombination repair pathway. This study was performed to detect the expressions of these two molecules in breast cancer and explore their correlations with clinicopathological factors.

**Methods and Results:**

Immunohistochemistry was used to detect protein expressions of XRCC3 and RAD51 in 248 cases of breast cancer tissue and 78 cases of adjacent non-cancerous tissue. Data showed that expressions for both XRCC3 and RAD51 were significantly increased in breast cancer. High XRCC3 expression was associated with large tumor size and positive PR and HER2 status, while high RAD51 expression was associated with axillary lymph node metastasis and positive PR and HER2 status. The result of multivariate analysis demonstrated that HER2, PR and RAD51 were significantly association with XRCC3. And besides XRCC3, axillary lymph node metastasis and PR were significantly correlated with RAD51.

**Conclusions:**

XRCC3 and RAD51 were significantly associated with clinicopathological factors and they might play important roles in the development and progress of breast cancer.

## Introduction

Breast cancer is one of the most common cancers and the leading cause of tumor-related death among women worldwide. Though the exact etiology remains unknown, increasing evidence indicates that breast cancer pathogenesis is tightly linked with double-strand break (DSB) repair dysfunction [1], [2].

RAD51, which catalyses strand transfer between a broken sequence and its undamaged homologue to allow re-synthesis of the damaged region, represents the central recombinase of homologous recombination repair (HRR). However, its localization to DSBs depends on the function and its direct interaction with XRCC3 [3], a RAD51 paralog that participates in the HRR pathway. It is known that RAD51 expression is significantly increased in breast cancer [4], [5]. And the research conducted by Maacke et al. suggested a correlation between wild-type RAD51 expression and histological grading invasive ductal breast cancer [4]. Though later study performed by Barbano et al. didn’t confirm this association, they found that high RAD51 mRNA expression was associated with breast cancer patient’s outcome [5]. Taking the similarity and close association between XRCC3 and RAD51 into account, it is speculated that XRCC3 may also play an important role in the pathogenesis of breast cancer. Most studies on XRCC3 were focused on its gene polymorphisms. And epidemiological studies have demonstrated a correlation between gene polymorphisms of XRCC3 and breast cancer risk [6]–[8]. But the expression of XRCC3 in breast cancer was not well studied. In this study, immunohistochemistry was used to explore the prevalence of XRCC3 and RAD51 expression and their possible roles in breast cancer.

## Patients and Methods

### Ethics Statement

All of the tissue specimens used in this study were obtained with patient written informed consent and the Ethics Committee of Changhai Hospital granted approval for this measure as well as the research protocol.

### Study Subjects

All primary breast cancer patients who had undergone initial surgery at The First Affiliated Hospital of Second Military Medical University (Changhai Hospital, Shanghai, China) between January 2009 and June 2010 were identified, by reviewing electronic charts. Patients who represented other primary tumor site or received preoperative radiotherapy or chemotherapy were excluded. Finally, a total of 248 patients (median age, 54.7 years old; range, 31 to 84 years old) were enrolled in this study. The following variables were recorded: patient age at diagnosis, menopausal status, largest tumor diameter, number of lymph node metastasis, TNM stage (UICC), histology grade (Elston-Ellis grade), ER, PR, and HER2. The paraffin-embedded pathologic specimens from surgical resection of these patients were obtained from the archives of Department of Pathology. All these resection samples had a uniform fixation, dissection and processing protocol. In addition, 78 cases of adjacent non-cancerous tissues were collected.

### Tissue Microarray (TMA) and Immunohistochemistry (IHC)

To cover more tumor cells and represent the typical pathological changes, large core TMAs were used. Briefly, TMA blocks were constructed as follows: 1.5 mm diameter cylinders from the center of the tumor away from areas of ulceration and necrosis were punched from representative areas of a tissue block, and re-embedded into a recipient paraffin block in a defined position, using a tissue arraying instrument (Beecher Instruments, Sun Prairie, WI, USA). Then, TMA blocks were cut into 4-µm sections and processed for IHC. Antibodies were purchased from Sigma-Aldrich (St. Louis, MO, USA) and diluted in phosphate-buffered saline/0.1% bovine serum albumin. The XRCC3 (SAB4503092) antibody and the RAD51 (SAB1406364) antibody were used at 10 ug/ml and 2 ug/ml, respectively, overnight at 4°C. Immunostaining was performed using the Envision System with diaminobenzidine (Dako, Glostrup, Denmark). A negative control was obtained by replacing the primary antibody with a normal murine or rabbit IgG at the same dilutions.

### IHC Evaluation

Expressions of XRCC3 and RAD51 in the TMAs were evaluated by two individuals (N.W. and Y.J.W.), who were blinded to the clinicopathological data of these breast cancer patients, at 200× magnification light microscopy. Discrepancies were resolved by discussion between the two evaluators.

### Semi-quantitative Criteria

A semi-quantitative evaluation of XRCC3 and RAD51 positivity by IHC was performed using a method described as follows: the percentage of positive cells was divided into five grades (percentage scores): ≤10% (0), 11–25% (1), 26–50% (2), 51–75% (3), and >75% (4). The intensity of staining was divided into four grades (intensity scores): no staining (0), light brown (1), brown (2), and dark brown (3). Staining positivity was determined by the formula: overall scores = percentage score×intensity score. The total score ranged from 0 to 12, with low expression (0–8) and high expression (9–12).

HER2 IHC was evaluated according to the Dako scoring system [9]. A positive HER2 result was IHC staining of 3+ or 2+ with a positive fluorescent in situ hybridization (FISH) result [10]. And the Leake’s score was used for evaluation of ER and PR [11]. ER and PR positivity was taken as a score ≥3 [12].

### Statistic Analysis

The STATA 10.0 software was applied for statistical analysis. Associations between different variables were assessed by Pearson’s chi-square test. Multivariate analysis was evaluated by logistic regression analysis. *P*-values <0.05 were considered to be statistically significant in all of the statistical analyses.

## Results

### Staining of XRCC3 and RAD51 in Breast Cancers

**Figure 1:**
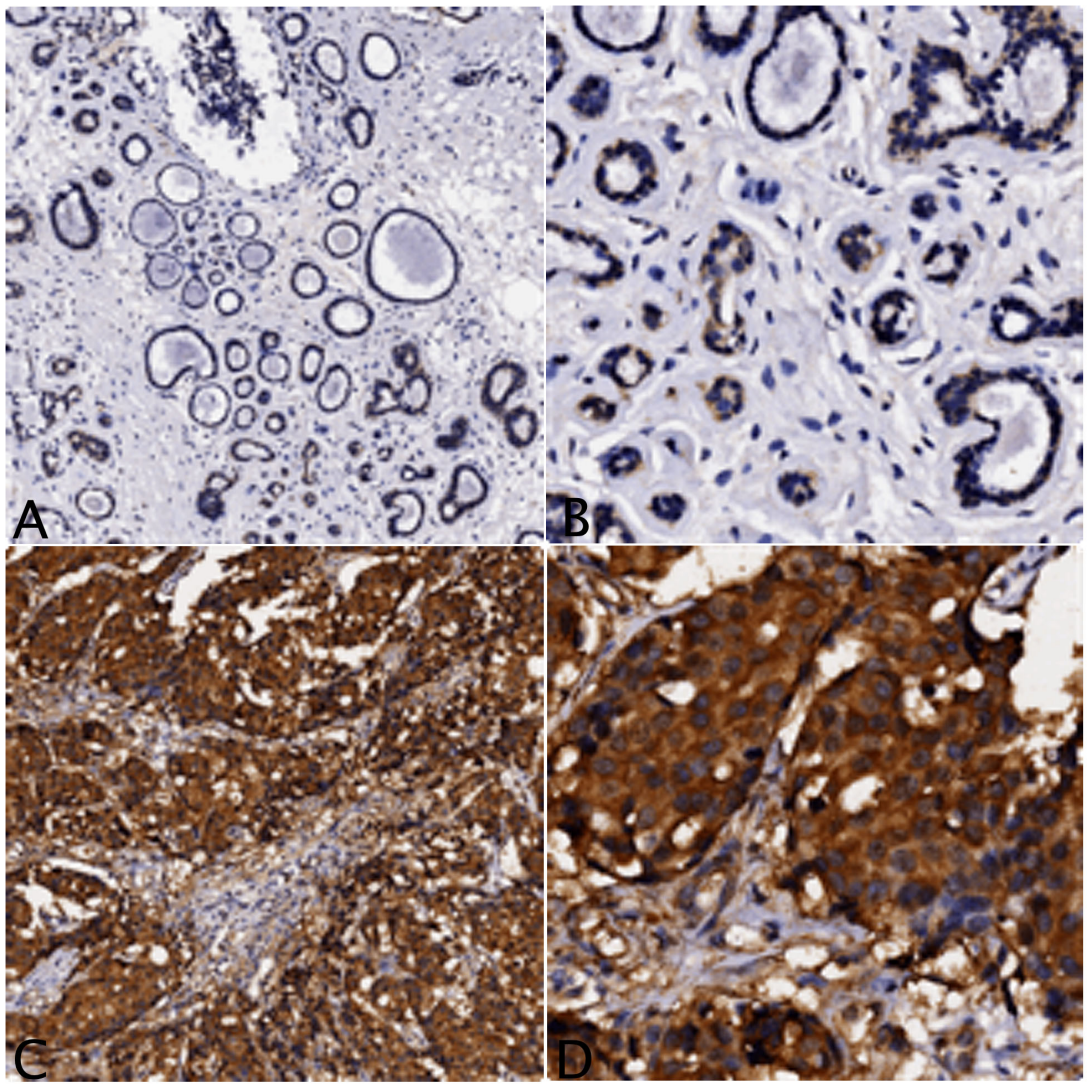
Representative results of XRCC3 protein expression by immunohistochemical analysis. Percentage of positive cells and staining intensity are much lower in adjacent non-cancerous tissue (A.×40; B.×200) than in breast cancer (C.×40; D.×200).

**Figure 2:**
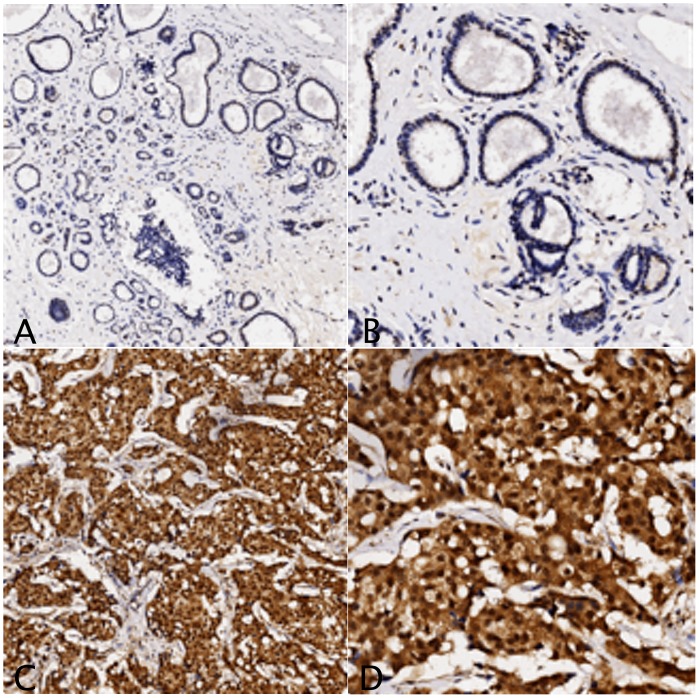
Representative results of RAD51 protein expression by immunohistochemical analysis. Percentage of positive cells and staining intensity are much lower in adjacent non-cancerous tissue (A.×40; B.×200) than in breast cancer (C.×40; D.×200).

Diffuse cytoplasmic and nuclear staining for XRCC3 (Figure 1) and RAD51 (Figure 2) was observed. As both XRCC3 and RAD51 are related with DNA repair and have to be active in the nucleus, only the nuclear staining was considered. The mean percentage of positive cells was much higher in breast cancers than in adjacent non-cancerous tissues (XRCC3: 64% vs. 20%; RAD51: 83% vs. 55%). Take staining intensity into consideration, nuclear staining for both XRCC3 and RAD51 was scored and subjected to statistical analysis. The results were summarized in Table 1. It suggested that expressions for both XRCC3 and RAD51 were significantly higher in breast cancer (XRCC3: *P*<0.001; RAD51: *P*<0.001).

**Table 1:** Expression of XRCC3 and RAD51 in breast cancer and adjacent non-cancerous tissue.

	XRCC3 expression		*Χ^2^/P* value	RAD51 expression		*Χ^2^/P* value
	Low	High		Low	High	
Adjacent non-cancerous tissue	76	2	**27.791**/	60	18	**63.340**/
Breast cancer	168	80	**<0.001**	66	182	**<0.001**

### Correlation to Clinicopathological Factors

Results on the association between XRCC3 and RAD51 expressions and some clinicopathological factors are presented in Table 2. Significant differences of XRCC3 expression were found between the classifications of subgroups of tumor size (*P* = 0.036), PR status (*P* = 0.031), and HER2 status (*P* = 0.034). RAD51 expression was significantly different according to the status of axillary lymph node metastasis (*P* = 0.004), as well as PR status (*P* = 0.011) and HER2 status (*P* = 0.036). And as expected, a strong association exists between XRCC3 expression and RAD51 expression (Table 3; *P*<0.001).

**Table 2:** Correlation between expression of XRCC3 and RAD51 and clinicopathological factors.

	XRCC3 expression		RAD51 expression		Total
	Low	High	Low	High	
Age at diagnosis					
≤45	31	22	12	41	53
>45	137	58	54	141	195
*Χ^2^*/*P* value	2.640/0.104		0.544/0.461		
Menopausal status					
Premenopausal	60	35	27	68	95
Postmenopausal	96	40	29	107	136
Unknown					17
*Χ* ^2^/*P* value	1.408/0.235		1.534/0.215		
Histology grade					
I–II	117	63	46	134	180
III	51	17	20	48	68
*Χ^2^*/*P* value	2.259/0.133		0.376/0.540		
Tumor size					
T1: ≤2 cm	63	25	29	59	88
T2: 2–5 cm	96	43	33	106	139
T3: >5 cm	9	12	4	17	21
*Χ^2^*/*P* value	**6.659/0.036**		3.015/0.222		
Axillary lymph node metastasis					
No	94	40	45	89	134
Yes	68	33	17	84	101
Unknown					13
*Χ^2^*/*P* value	0.214/0.643		**8.312/0.004**		
TNM stage					
1	37	12	19	30	49
2	95	40	34	101	135
3	30	21	9	42	51
Unknown					13
*Χ^2^*/*P* value	3.554/0.169		5.978/0.050		
ER					
–	58	27	22	63	85
+	110	53	44	119	163
*Χ^2^*/*P* value	0.014/0.904		0.035/0.851		
PR					
–	70	22	33	59	92
+	98	58	33	123	156
*Χ^2^*/*P* value	**4.661/0.031**		**6.417/0.011**		
HER2					
–	125	49	53	121	174
+	43	31	13	61	74
*Χ^2^*/*P* value	**4.480/0.034**		**4.419/0.036**		

**Table 3:** Association between XRCC3 and RAD51.

RAD51	XRCC3		Total	*Χ^2^*	*P* value
	Low	High			
Low	60	6	66	**22.089**	**<0.001**
High	108	74	182		

### Multivariate Logistic Regression Analysis

Because TNM stage was dependent on tumor size and axillary lymph node metastasis, it was excluded in multivariate analysis. Variables that were selected for multivariate analysis included XRCC3, RAD51, HER2, ER, PR, age at diagnosis, histology grade, tumor size, and axillary lymph node metastasis. The values assigned to these variables were as follows: age at diagnosis ≤45 years old = “1”, >45 years old = “2”; histology grade: I = “1”, II = “2”, III = “3”; tumor size: ≤2 cm = “1”, 2.1–5 cm = “2”, >5 cm = “3”; axillary lymph node metastasis: 0 = “1”, 1–3 = “2”, 4–9 = “3”, >10 = “4”; XRCC3, RAD51, ER, PR, HER2: low expression or negative = “1”, high expression or positive = “2”.

XRCC3 and RAD51 were selected as dependent variable to perform multivariate analysis respectively. As Table 4 showed, HER2, PR and RAD51 were demonstrated a significant association with XRCC3. And XRCC3, axillary lymph node metastasis and PR were significantly correlated with RAD51.

**Table 4:** Multivariate analysis by logistic regression analysis.

Dependent variable	Independentvariable	Regression coefficient	SE	*P* value	OR	95% CI
XRCC3	HER2	0.703	0.352	0.046	2.019	1.012–4.028
	PR	0.758	0.369	0.040	2.133	1.035–4.395
	RAD51	1.753	0.504	0.001	5.773	2.151–15.494
RAD51	Axillary lymph node metastasis	0.431	0.200	0.031	1.539	1.040–2.278
	PR	0.859	0.367	0.019	2.361	1.150–4.848
	XRCC3	1.777	0.504	<0.001	5.914	2.203–15.881

## Discussion

As a major defense against environmental damage to cells, DNA repair is vital to the integrity of genome. Abnormality in this process is believed to implicate in tumorigenesis. Theoretically, decreased or loss of expression of DNA repair proteins could damage the DNA repair capacity and lead to genomic instability, thus increase susceptibility to cancer. However, a series of studies suggested that changes in the expression of DNA repair protein were quite complicated during tumorigenesis, as some were down-regulated but others were up-regulated[4], [5], [13]–[15]. XRCC3 is a human homolog of RAD51 and participate in the HRR pathway. It plays an important role in the assembly or stabilization of a multimeric form of RAD51 during DNA repair [3]. RAD51 was reported to have a significantly increased expression in immortalized and tumor cells, including breast cancer. While few studies on XRCC3 expression in breast cancer were reported. Our results showed that the expression levels of both XRCC3 and RAD51 were significantly increased in breast cancer, which was consistent with their high mRNA expressions. How to explain this phenomenon? Is it a result of cells responding to DNA damage but not sufficient to maintain the genome stability? Or the overexpression of these proteins promotes genome instability and tumorigenesis? Richardson et al. used a genetic system to examine the potential for multiple DSBs to lead to genome rearrangements in the presence of increased RAD51 expression, and found a connection between elevated RAD51 protein levels and genome instability as well as tumor progression [16]. Studies on XRCC3 function revealed that XRCC3 was required for the proliferation of MCF7 cells and the decrease in its expression leaded to the accumulation of DNA breaks and the induction of p53-dependent cell death [17]. And on the other hand, cells over-expressing XRCC3 were more invasive and showed a higher tumorigenesis in vivo [18]. What’s more, our study suggested significant associations existed between the expression of these two markers and HER2 level, which was a strong poor prognostic factor in breast cancer. And high expression of XRCC3 and RAD51 were associated with large tumor size and axillary lymph node metastasis respectively. Base on these results, we are disposed to agree that overexpression of XRCC3 and RAD51 may play an important role in the pathogenesis of breast cancer.

Accumulated evidence indicated that activation of erbB family of receptors could promote chemo- and radiotherapy resistance when mutated or over-expressed [19]–[21]. And recent studies demonstrated a role of erbB-signaling in regulating DSB repair [22]–[26]. Not only did it regulate the DNA repair capacity through immediate activation of downstream pathways that might control the fast component of DNA-DSB repair [25], but also it was involved in activating DNA-DSB repair through its translocation to the nucleus, which might be important for the slow component of DNA-DSB [26]. Golding et al. found that expression of EGFR variant III could increase the formation of phospho-DNA-PKcs and –ATM repair foci, and RAD51 foci and expression levels, while expression of dominant-negative EGFR exerted an opposite effect [22]. In this study, we found that a significant correlation existed between HER2 and the expression of XRCC3 and RAD51, indicating an effect of HER2 on the transcription of genes that coded for proteins involved in the repair of DNA damages.

Previous studies suggested that progesterone had an affect on DNA repair ability [27]–[29]. But the relationship between PR and DNA repair was seldom studied. In this study, our data suggested positive PR status was significantly associated with high expression of XRCC3 and RAD51, even in multivariate analysis. Interestingly, Barbano et al. explored the expression of RAD51 in breast cancer and observed that RAD51 expression was inversely associated with PR status [5]. Despite the contrary result, PR was suggested a potential role in DNA repair. But the exact mechanism needs further investigation.

XRCC3 and RAD51 are two important molecules in HRR pathway. Laboratory researches found that abnormalities in their function were not only associated with tumorigenesis, but also metastasis and chemo- and radiotherapy resistance [18], [30], [31]. In this study, we used clinical specimens and confirmed the role of XRCC3 and RAD51 in development and progress of breast cancer. AND the significant correlation among the expressions of PR, HER2, XRCC3 and RAD51 indicated that PR and HER2 might be involved in DNA repair. The further mechanism research will not only help clarify the etiology of breast cancer, but also provide effective means to prevent tumor metastasis and resolve the problems of tumor resistance.
